# STAT2 Knockout Syrian Hamsters Support Enhanced Replication and Pathogenicity of Human Adenovirus, Revealing an Important Role of Type I Interferon Response in Viral Control

**DOI:** 10.1371/journal.ppat.1005084

**Published:** 2015-08-20

**Authors:** Karoly Toth, Sang R. Lee, Baoling Ying, Jacqueline F. Spencer, Ann E. Tollefson, John E. Sagartz, Il-Keun Kong, Zhongde Wang, William S. M. Wold

**Affiliations:** 1 Department of Molecular Microbiology and Immunology, Saint Louis University School of Medicine, St. Louis, Missouri, United States of America; 2 Department of Animal, Dairy and Veterinary Sciences, Utah State University, Logan, Utah, United States of America; 3 Department of Comparative Medicine, Saint Louis University School of Medicine, St. Louis, Missouri, United States of America; 4 Department of Animal Science, Division of Applied Life Science and Institute of Agriculture and Life Science, Gyeongsang National University, Jinju, Republic of Korea; University of Glasgow, UNITED KINGDOM

## Abstract

Human adenoviruses have been studied extensively in cell culture and have been a model for studies in molecular, cellular, and medical biology. However, much less is known about adenovirus replication and pathogenesis *in vivo* in a permissive host because of the lack of an adequate animal model. Presently, the most frequently used permissive immunocompetent animal model for human adenovirus infection is the Syrian hamster. Species C human adenoviruses replicate in these animals and cause pathology that is similar to that seen with humans. Here, we report findings with a new Syrian hamster strain in which the STAT2 gene was functionally knocked out by site-specific gene targeting. Adenovirus-infected STAT2 knockout hamsters demonstrated an accentuated pathology compared to the wild-type control animals, and the virus load in the organs of STAT2 knockout animals was 100- to 1000-fold higher than that in wild-type hamsters. Notably, the adaptive immune response to adenovirus is not adversely affected in STAT2 knockout hamsters, and surviving hamsters cleared the infection by 7 to 10 days post challenge. We show that the Type I interferon pathway is disrupted in these hamsters, revealing the critical role of interferon-stimulated genes in controlling adenovirus infection. This is the first study to report findings with a genetically modified Syrian hamster infected with a virus. Further, this is the first study to show that the Type I interferon pathway plays a role in inhibiting human adenovirus replication in a permissive animal model. Besides providing an insight into adenovirus infection in humans, our results are also interesting from the perspective of the animal model: STAT2 knockout Syrian hamster may also be an important animal model for studying other viral infections, including Ebola-, hanta-, and dengue viruses, where Type I interferon-mediated innate immunity prevents wild type hamsters from being effectively infected to be used as animal models.

## Author Summary

The biology of human adenoviruses has been studied extensively; however, much less is known about the replication and pathogenesis of the virus in a permissive host. Our laboratory pioneered the use of Syrian hamsters to study the pathogenesis of human adenoviruses. Syrian hamsters are permissive for species C human adenoviruses, which replicate in these animals and cause illness akin to that in humans. Hereby, we report findings with a new Syrian hamster strain (STAT2 KO hamsters), in which the Type I interferon pathway, an important part of the innate immune response to virus infection, is disrupted. This is the first genetically modified Syrian hamster strain ever reported. We show that these animals are very sensitive to infection with type 5 human adenovirus (Ad5). Ad5 replicates to 100- to 1000-fold higher titers in STAT2 KO hamsters than in wild-type ones, and this increased infection causes enhanced pathology. However, the adaptive immune response to the virus infection seems to be intact with the STAT2 KO hamsters, and surviving animals clear the virus effectively. The data reported here may be of interest to researchers focusing on adenoviruses, and also to those who utilize the Syrian hamster as their animal model for other purposes.

## Introduction

Human adenoviruses (Ads) are non-enveloped, double-stranded DNA viruses that are classified into over 60 types (Ad1, Ad2, etc.), which in turn are grouped into 7 species (A to G) (for a review on Ad biology, see [1, 2]). Infection with the more frequent types is very common; about 40 to 60% of children are seropositive for Ad1, 2, or 5 [3, 4]. Ads cause a variety of diseases in humans; the symptoms range from respiratory to enteric, ocular and urinary, depending on the type of Ad. Generally, in immunocompetent adults the course of the infections is mild, and it resolves without the need for medical intervention (for a review of Ad epidemiology and pathology, see [3, 5]). Infection with a specific type causes long-term immunity to that type.

In certain circumstances, Ads can cause serious illness. Some types are more pathogenic than others: Ad8 and other types can cause epidemic keratoconjunctivitis (EKC), a disease that can result in lasting vision defects, even blindness. Other Ad diseases are more dependent of the health status of the host: in military recruits, Ad4, 7, 3, 21, and 14 cause serious acute respiratory disease that in some cases can lead to death. The most extreme case of the host’s influence on the illness caused by Ads is seen with immunocompromised patients. For these patients, the same types that cause self-resolving illness in healthy adults can cause serious, often life-threatening illness. The reason is that the dysfunctional immune system of these people cannot clear the primary virus infection, and thus it can develop into a much more severe systemic disease. In allogeneic hematopoietic stem cell transplant patients with a rising load of Ad in peripheral blood (as determined by quantitative PCR [qPCR]) despite antiviral therapy, the mortality can approach 100% [3, 6, 7].

The host’s response to Ad infection has been extensively studied in cell culture and *in vivo;* the *in vivo* studies have been nearly always performed using non-permissive mice, usually with replication-defective Ad vectors. It was demonstrated that Ad infection induces vigorous innate immune response (for a recent review, see [8]). As part of the innate immune response, Ad infection results in the production of Type I interferons (IFNs). The production of IFNα and IFNβ by a variety of mononuclear cells was demonstrated in cell culture [9, 10].

In recent studies in cell culture, cGAS/STING was shown to be the sensor for Ad DNA in the cytoplasm leading to the IFN response [11, 12]. With this sensor, upon DNA binding, cyclic GMP-AMP synthetase synthesizes cyclic guanine-adenine dinucleotide (cGAMP). cGAMP binds to the STING adaptor protein, which competes with TANK-binding kinase leading to phosphorylation of IFN response factor 3 (IRF3). IRF3 translocates to the nucleus to induce transcription of IFN and IFN-stimulated genes (ISGs). These Ad studies were conducted with murine endothelial and Raw264.7 macrophage lines infected with replication-defective Ad vectors [12] and with various permissive human cell lines infected with wild-type (wt) Ad [11]. It is not known whether Syrian hamsters have this DNA sensor.

*In vivo* in mice, myeloid dendritic cells in the spleen were shown to be the most prolific producers of Type I IFNs [9]. In these cells, the induction of IFNα and IFNβ is mediated by a hitherto unknown cytosolic sensor [9]. This seems to be unique, because plasmacytoid dendritic cells are reported to produce most of Type I IFNs during other virus infections, and the induction is mediated by a TLR9-dependent pathway, signaling via IRF7 [13]. In addition, different types of Ad seem to have different potential to induce a Type I IFN response [14], and this differential induction can lead to differential activation of NK cells [15]. These discrepancies are confounded by the fact, as mentioned, that the mouse is not an adequate model for studying human Ads, inasmuch as human Ads replicate poorly in mice. Thus, immune responses initiated by actual virus replication cannot be studied in mice.

Our laboratory and others have pioneered the use of Syrian hamsters as an animal model to study the replication and pathogenesis of human species C Ads *in vivo*, as well as a model system to investigate oncolytic Ad vectors. Species C human Ads replicate in most organs of Syrian hamsters after intranasal or intravenous injection, and cause pathology that is similar to that seen with humans [16–29]. For immunocompetent hamsters, the immune response clears Ad infections by approximately 1 week post challenge, and without exception the animals recover from the infection [28, 29]. Hamsters immunosuppressed by high doses of cyclophosphamide are unable to eliminate the infection, and develop enhanced pathology, mirroring the pathology caused by Ad infections of immunocompromised patients [30]. We have shown that the immunosuppressed Syrian hamster model can be used to test the efficacy of anti-adenoviral drugs *in vivo* [30–33]. In the latter studies we have shown that brincidofovir (formerly named CMX001), cidofovir, ganciclovir and valganciclovir are very effective both prophylactically and therapeutically in treating disseminated Ad5 infections.

With several studies mentioned above, cyclophosphamide was used to immunosuppress the animals. However, there are some significant drawbacks in using cyclophosphamide, chief among them is that it exerts pleiotropic effects. Recently, a Syrian hamster strain with the STAT2 gene inactivated was generated with CRISPR/Cas9-mediated site-specific gene targeting, and the authors showed that STAT2 knockout (STAT2 KO) hamsters do not express the full length STAT2 protein [34]. STAT2 is a crucial element of the Type I and Type III IFN signal transduction pathway (reviewed in [35, 36]). STAT2-null mice are defective in inducing ISGF3 target genes and are more susceptible to viral infections [37]. Here, we demonstrate that the Type I IFN pathway is disrupted in STAT2 KO hamsters, which makes these animals extremely sensitive to intravenous infection with Ad5. At 3 days post challenge, the virus burden in the organs of STAT2 KO animals is 1000-fold higher than in wt hamsters. Our results underscore the importance of innate responses in fighting Ad infections, and may provide relevant information to the management of Ad infections in immunocompromised patients.

## Results

### Intravenous infection with Ad5 causes increased pathogenicity with STAT2 KO hamsters compared to wt animals

**Fig 1:**
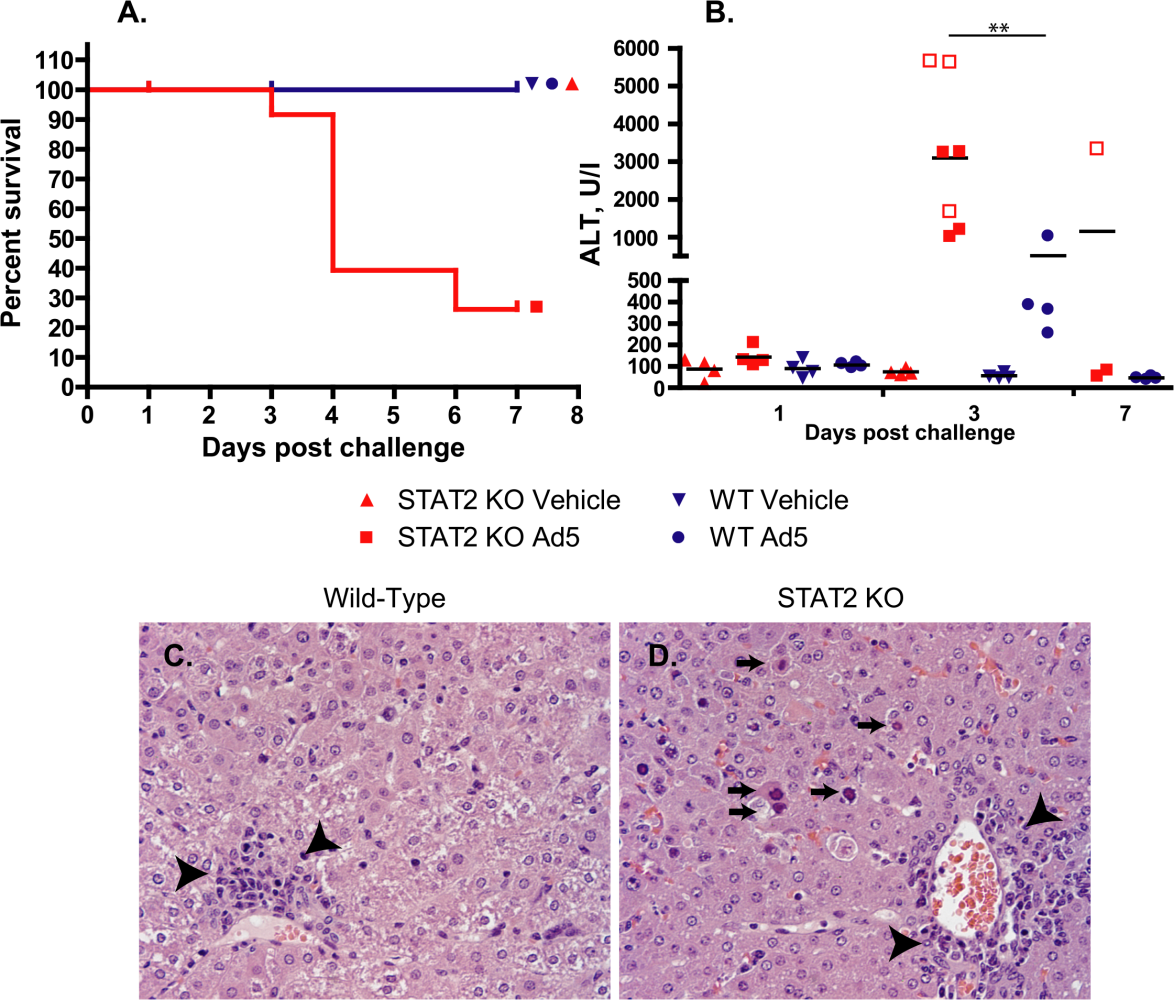
Ad5 infection causes increased pathology with STAT2 KO hamsters compared to wt ones. **A.** Survival. wt Ad5 v. STAT2 KO Ad5 p = 0.0024 (Log rank test) **B.** Serum alanine aminotransferase levels. Symbols denote data from individual animals; empty symbols signify that the samples were collected from an animal that was sacrificed moribund. **: P<0.01 Liver histopathology at 3 days post infection for wt (**C**) and STAT2 KO (**D**) hamsters. The arrows indicate hepatocellular necrosis; the arrowheads point at areas with mononuclear infiltration.

Sixteen STAT2 KO and 16 wt hamsters were injected with Ad5 as described in the Methods section. Four animals of each strain were scheduled to be sacrificed at 1 and 3 days post infection (p.i.), and 8 hamsters were scheduled to be sacrificed at 7 days p.i. However, 6 of the 8 Day 7 STAT2 KO hamsters were moribund at around 4 days p.i. (Fig 1A). These animals were sacrificed, and for some subsequent analyses their data were grouped with those of animals sacrificed according to schedule at 3 days p.i. In figures depicting such combined results, data obtained from moribund animals is discernible from data gathered during scheduled sacrifice.

Altogether, there were 6 treatment-related deaths in this study, all in the Ad5-infected STAT2 KO group (Fig 1A). For the animals killed at the scheduled time point at 3 days p.i., gross pathology revealed that 2 of the 4 Ad5-injected STAT2 KO hamsters had pale, mottled liver, while no significant findings were noted for the other two hamsters. The hamsters that were sacrificed moribund (all STAT2 KO) presented pathology characteristic of advanced Ad infection (yellow, mottled liver, enlarged gall bladders). No pathology was observed with the wt hamsters at any sacrifice time point. Histopathological analysis of the liver confirmed that the peak of pathology was at 3 to 4 days post challenge (Table 1 and Fig 1C). Ad5-infected STAT2 KO hamsters sacrificed according to schedule at this time showed moderate multifocal mononuclear infiltration, multifocal hepatocellular necrosis of minimal to mild severity and, marked diffuse decreased vacuolation. The pathology was even more obvious with the hamsters that were sacrificed moribund at 3 and 4 days post challenge: these animals presented with moderate to marked hepatocellular necrosis with nuclear inclusion bodies, which is characteristic of Ad5 infections of the liver [28, 38]. At 3 days post challenge, the only significant findings for the Ad5-infected wt hamsters were mild to moderate decreased vacuolation and minimal to mild mononuclear infiltration (Table 1 and Fig 1C). At 7 days post challenge, minimal mononuclear infiltration and marked decreased vacuolation was reported for the surviving STAT2 KO hamsters, while no significant microscopic lesions were found in the liver of wt animals (Table 1). A STAT2 KO animal that was sacrificed moribund at 6 days post challenge had lesions of similar nature and severity as the animals sacrificed moribund at 3 to 4 days post challenge.

**Table 1:** Histopathological findings in the liver.

Strain	Treatment	Finding	Distribution	Severity^1^	Number of Animals Affected
**Wild**	Vehicle	NSML^2^			8/8
**Type**	Ad5, Day 1	Inflammation/Infiltration	focal	0.25 (0–1)	1/4
	Ad5, Day 3	Inflammation/Infiltration	multifocal	1.5 (1–2)	4/4
		Decreased Vacuolation	diffuse	2.75 (2–3)	4/4
	Ad5, Day 7	NSML			8/8
**STAT2**	Vehicle	Inflammation/Infiltration	multifocal	0.375 (0–1)	3/8
**KO**		Decreased Vacuolation	diffuse	0.5 (0–4)	1/8
	Ad5, Day 1	Inflammation/Infiltration	multifocal	1.5 (1–2)	4/4
		Decreased Vacuolation	diffuse	2 (1–3)	4/4
	Ad5, Day 3	Inflammation/Infiltration	multifocal	3 (3)	4/4
		Decreased Vacuolation	diffuse	4(4)	4/4
		Hepatocellular Necrosis	multifocal	1.75(1–2)	4/4
	Ad5,	Inflammation/Infiltration	multifocal	3.4 (3–4)	5/5
	Day 3 MB^3^	Decreased Vacuolation	diffuse	4(4)	5/5
		Hepatocellular Necrosis	multifocal	3.4(2–4)	5/5
	Ad5, Day 7	Inflammation/Infiltration	multifocal	1 (1)	2/2
		Decreased Vacuolation	diffuse	4(4)	2/2
	Ad5,	Inflammation/Infiltration	multifocal	3	1/1
	Day 7 MB^3^	Decreased Vacuolation	diffuse	4	1/1
		Hepatocellular Necrosis	diffuse	4	1/1

^1^ Group mean (Range); 0: No lesions, 1 = Minimal; 2 = Mild; 3 = Moderate; 4 = Marked

^2^ No significant microscopic lesions

^3^ Sacrificed moribund ahead of schedule

Consistent with these observations, the Ad5-infected STAT2 KO animals had significantly higher serum alanine transaminase (ALT) levels than wt ones at 3 days post challenge (Fig 1B). By 7 days post challenge, ALT levels returned to normal levels in all surviving animals.

### The virus load is higher in the liver of STAT2 KO hamsters than in the liver of wt animals

**Fig 2:**
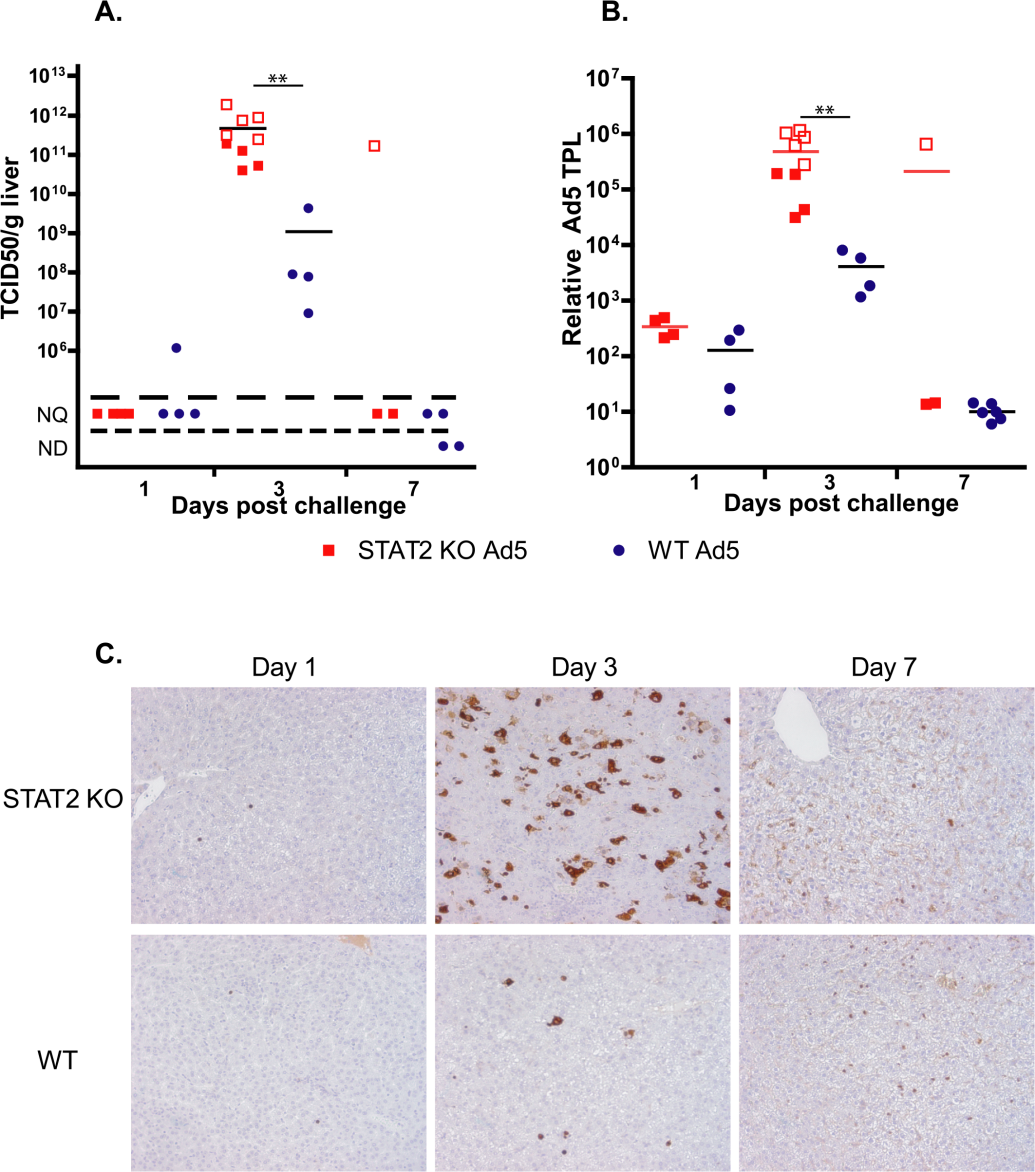
The virus load is higher in Ad5-infected STAT2 KO hamsters than in wt animals. **A.** Infectious virus load in the liver. **B.** Rate of active infection (expression of adenoviral tripartite leader (TPL)-containing late adenoviral mRNA; expressed as fold increase). For **A** and **B**, symbols denote data from individual animals; empty symbols signify that the samples were collected from an animal that was sacrificed moribund. **: P<0.01 **C.** Immunohistochemical staining for adenoviral fiber.

**Fig 3:**
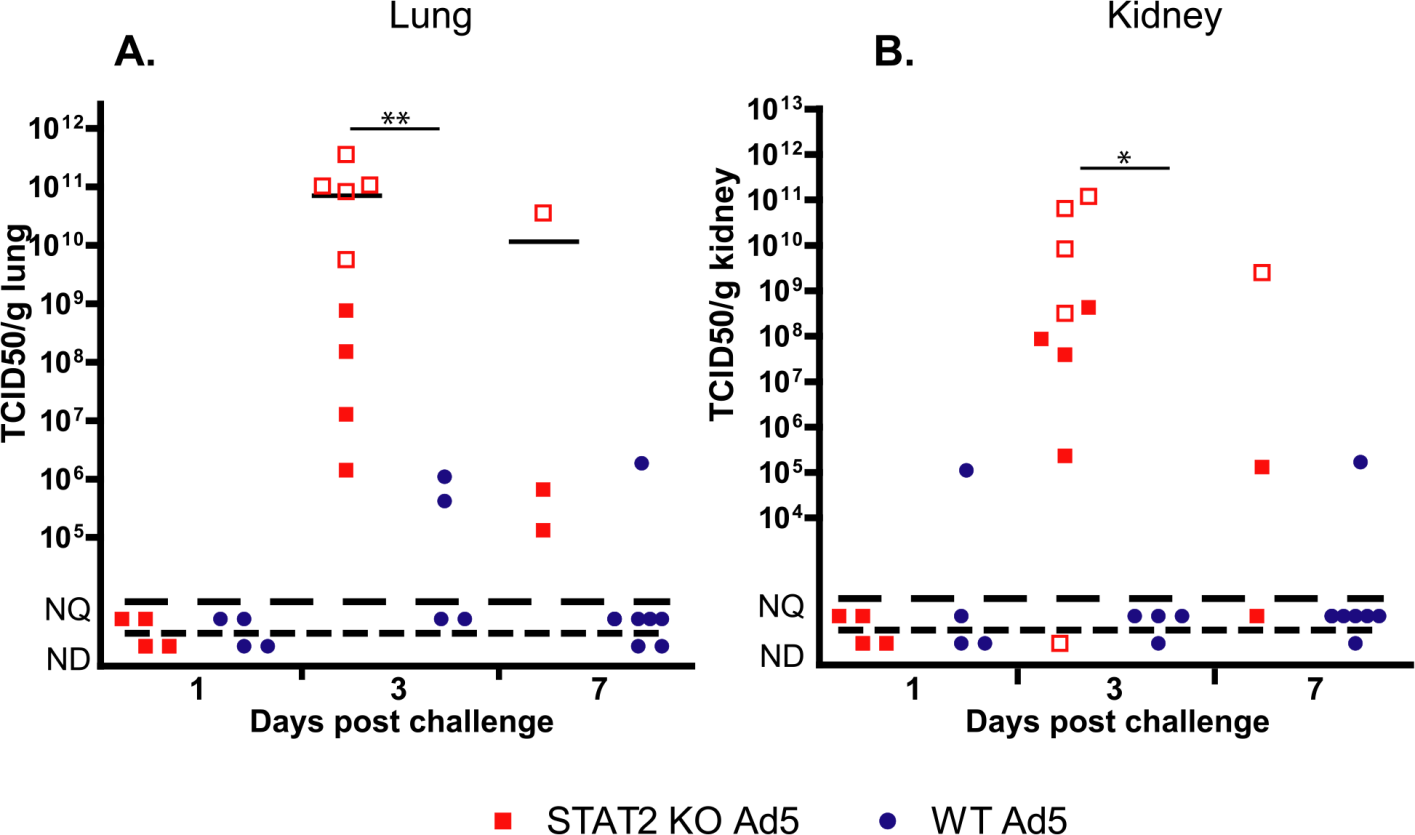
The infectious virus load in the lung (A) and kidney (B) of STAT2 KO hamsters is elevated after intravenous infection with Ad5 compared to wt hamsters. Symbols denote data from individual animals; empty symbols signify that the samples were collected from an animal that was sacrificed moribund.

Liver samples were collected at necropsy and were analyzed for infectious virus burden using a cell culture assay. At 3 days p.i., STAT2 KO hamsters had 100- to 1000-fold higher virus load in their liver than wt animals (Fig 2A). By Day 7, all surviving animals had undetectable to unquantifiable levels of virus load in the liver. To ascertain whether this elevation in virus load at 3 days p.i. was the result (of enhanced virus replication or delayed clearance of the released progeny virus, we determined the relative number of late adenoviral transcripts in the isolated liver samples as a surrogate for ongoing Ad replication. Ad late transcripts were measured by RT-qPCR using primers that are specific to the tripartite leader present in most mRNAs in the major late transcription unit. We found that there were a 100- to 1000-fold more late viral mRNA copies in samples collected from STAT2 KO animals than in samples from wt ones (Fig 2B), indicating that the increased virus load was the result of increased virus replication in the liver of STAT2 KO hamsters. Immunohistochemical staining for Ad fiber, a late Ad protein, corroborated the findings depicted in Fig 1A and 1B, inasmuch there were markedly more cells staining for the Ad fiber protein in the liver of STAT2 KO hamsters than in the liver of wt animals at 3 days p.i. (Fig 2C). The multifocal distribution of hepatocytes staining for Ad fiber (Fig 2C) coincides with the multifocal distribution of hepatocellular necrosis found during histopathological examination (Fig 1C). This finding suggests that Ad5 is the causative agent of the pathology observed in these animals. Most remarkably, when we analyzed the lung and the kidney for infectious virus load, we found that the these organs from STAT2 KO animals had virus load commensurate to the liver, while only a very low virus load was detectable in the lung and kidney of wt hamsters (Fig 3).

### The Type I IFN response is impaired with STAT2 KO hamsters

**Fig 4:**
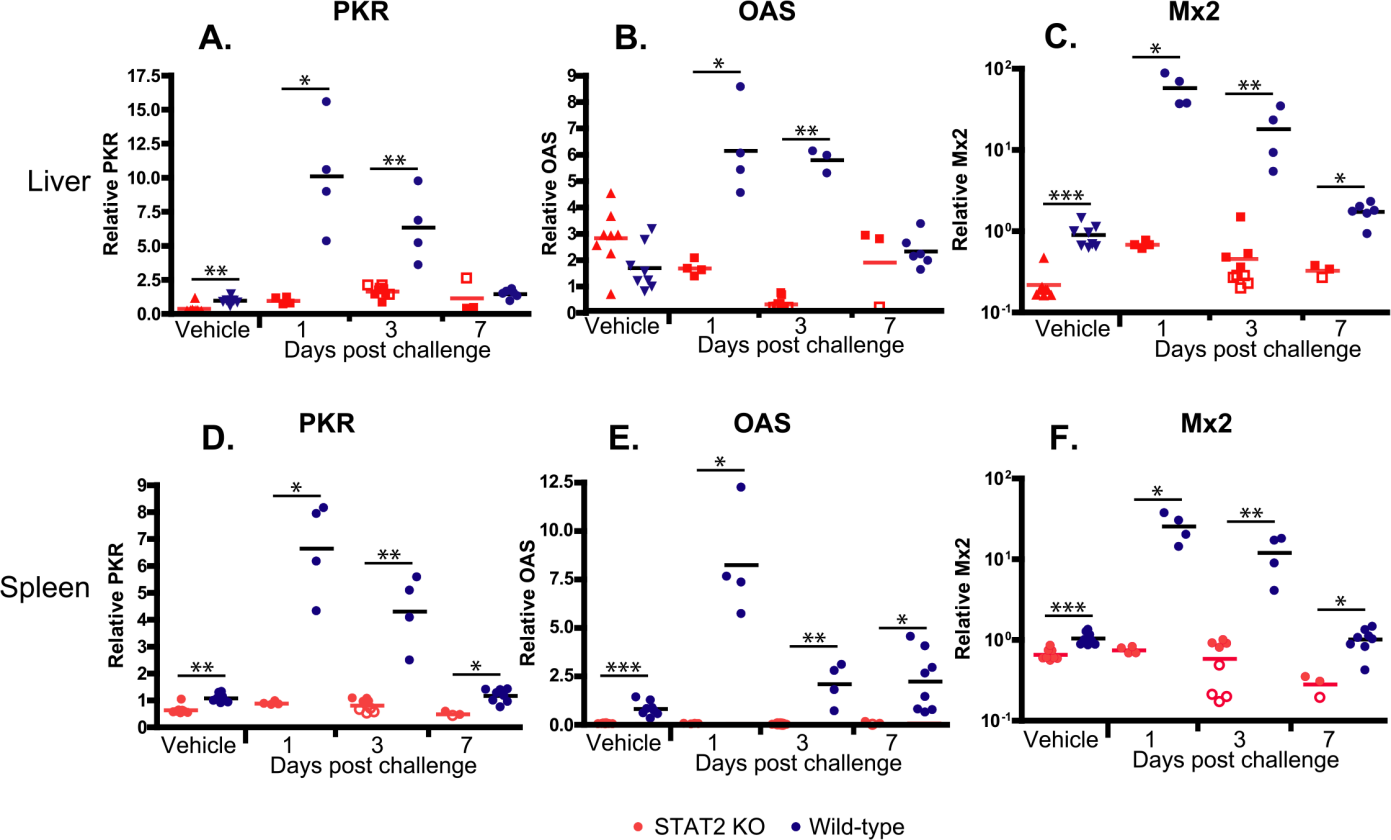
STAT2 KO hamsters fail to up-regulate the expression of interferon-stimulated genes PKR (A, D), OAS (B, E), and Mx2 (C, F) in the liver (A-C) and spleen (D-F). Fold changes in mRNA levels are shown; symbols denote data from individual animals; empty symbols signify that the samples were collected from an animal that was sacrificed moribund. *: P<0.05; **: P<0.01; ***: P<0.001.

If the Type I IFN response is impaired in the STAT2 KO hamsters, then we would expect that transcription of IFN-stimulated genes (ISGs) would be impaired. RT-qPCR was utilized to detect changes in the expression levels of ISGs in the liver, namely protein kinase R (double-stranded RNA-dependent protein kinase, PKR), oligoadenylate synthetase (OAS), and IFN-induced GTP-binding protein (Mx2). Baseline levels of PKR and Mx2 were significantly elevated in the livers of wt hamsters compared to the STAT2 KO animals (Fig 4A and 4C). The difference was much more striking after Ad5 infection: for the wt hamsters’ transcripts, all three ISGs assayed were extremely elevated at 1 and 3 days post challenge, while no such elevation was observed for the STAT2 KO animals (Fig 4A–4C). The induction of ISGs was similarly impeded in the spleen (Fig 4D–4F).

**Fig 5:**
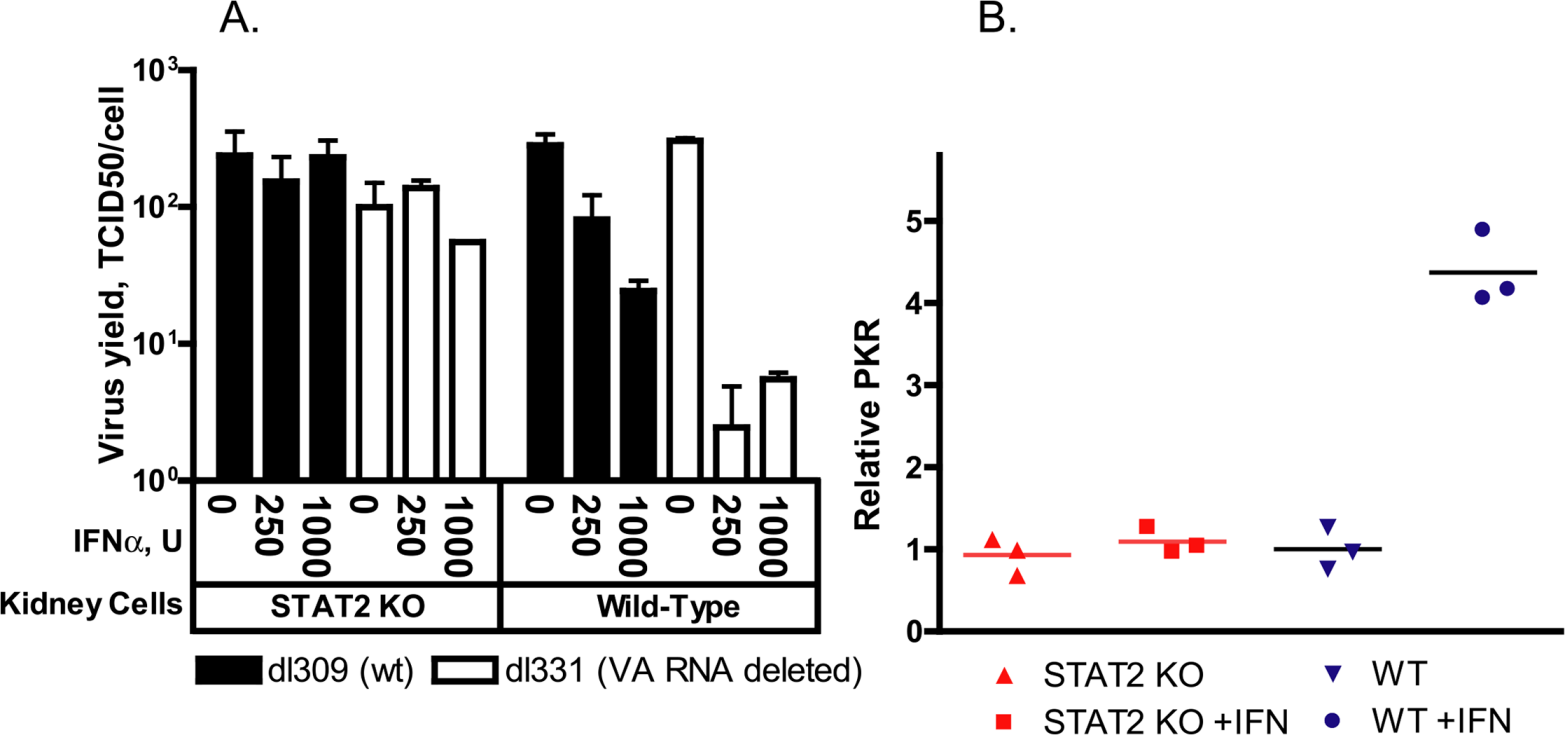
IFNα treatment of primary kidney cells derived from wt but not from STAT2 KO hamsters inhibits the replication of adenovirus. **A.** The average of infectious virus yield from two parallel experiments is shown. **B.** Induction with 250 U of human IFNα results in elevated PKR mRNA levels in wt but not in STAT2 KO primary kidney cells. Individual data from three biological replicates are shown.

To assess whether disruption of the Type I IFN pathway contributes to increased Ad replication in cell culture, we prepared primary kidney cultures of wt and STAT2 KO hamsters. Primary kidney cells were used because Ads replicate in hamster kidneys *in vivo*, thus these cells of epithelial origin are expected to support Ad replication in *vitro*. Further, established protocols exist for preparing such cells from harvested hamster kidneys. We infected both kidney cultures with either a mutant Ad named *dl*331 that is deleted for the VA RNA I sequence or with the parental Ad (*dl*309) in which the VA RNA I gene is intact. VA RNA I is a small Ad-coded RNA that counteracts the effect of PKR in Ad-infected cells [39], and it is required for the efficient translation of viral RNAs at the late stage of infection [40]. In human cells, the deletion mutant *dl*331 is very susceptible to IFN-mediated inhibition of virus replication, while *dl*309 is somewhat resistant [39]. When Ad-infected wt hamster kidney cells were treated with human IFNα, which is known to be active in Syrian hamsters [41], the yields of *dl*331 decreased approximately 100-fold, while there was a more modest but detectable decrease in the yields of *dl*309 (Fig 5A). However, no reduction in the yields of either virus was seen in IFNα-treated STAT2 KO kidney cells (Fig 5A). When we tested for the induction of ISGs in human IFNα-treated primary kidney cells, we found that PKR was efficiently induced in wt but not in STAT2 KO cells (Fig 5B), confirming that human IFNα activates the Type I IFN pathway in hamster cells. These data indicate that the disrupted Type I IFN pathway of the infected cells contributes to the increased Ad replication in the STAT2 KO animals. The data also indicate that the VA RNA I of human Ad5 is functional in primary hamster kidney cells.

**Fig 6:**
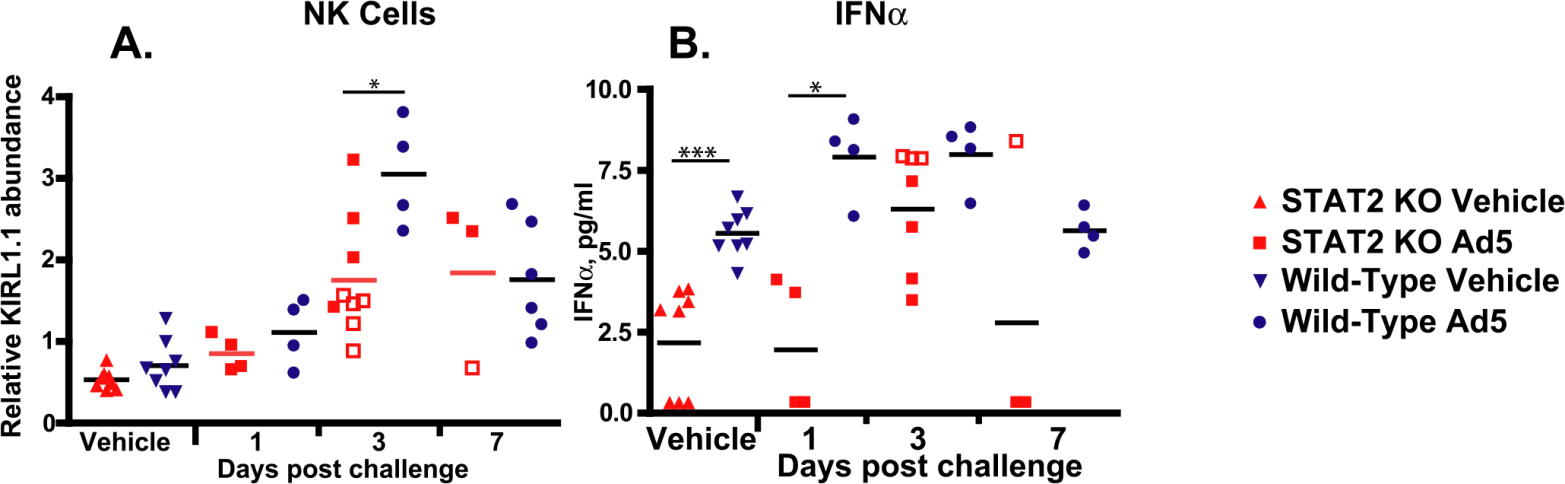
STAT2 KO hamsters have a blunted natural killer cell response (A), and delayed production of IFNα (B). Fold changes in mRNA levels are shown; symbols denote data from individual animals; empty symbols signify that the samples were collected from an animal that was sacrificed moribund. *: P<0.05; **: P<0.01; ***: P<0.001.

Besides affecting the antiviral state of the infected cell, Type I IFNs can have an indirect antiviral effect by modulating the functions of other immune cells [42]. One such cell population is natural killer (NK) cells, the cytotoxic function of which is increased by Type I IFN [43]. At 3 days post challenge, infiltration of the liver by NK cells was significantly reduced in STAT2 KO hamsters compared to wt animals, as judged by the changes in the abundance of mRNAs for KIRL1.1, an NK cell marker (Fig 6A). The decrease in KIRL1.1 expression was particularly marked in animals sacrificed moribund.

We also examined if obstructing the Type I IFN signaling pathway influences the expression of IFNα, inasmuch as it is known that the expression of IFNα depends on a virtuous cycle maintained by paracrine stimulation [37]. We found that there was a significant delay in the increase of IFNα serum concentration with the STAT2 KO hamsters. The baseline concentration of IFNα in the serum was significantly lower in these hamsters and did not reach the concentration of IFNα seen in the serum of wt hamsters until 3 days post challenge (Fig 6B). There was no statistically significant difference between the maximal IFNα concentrations reached in the two stains of hamsters (Fig 6B).

### The adaptive immune response of STAT2 KO hamsters is largely normal

**Fig 7:**
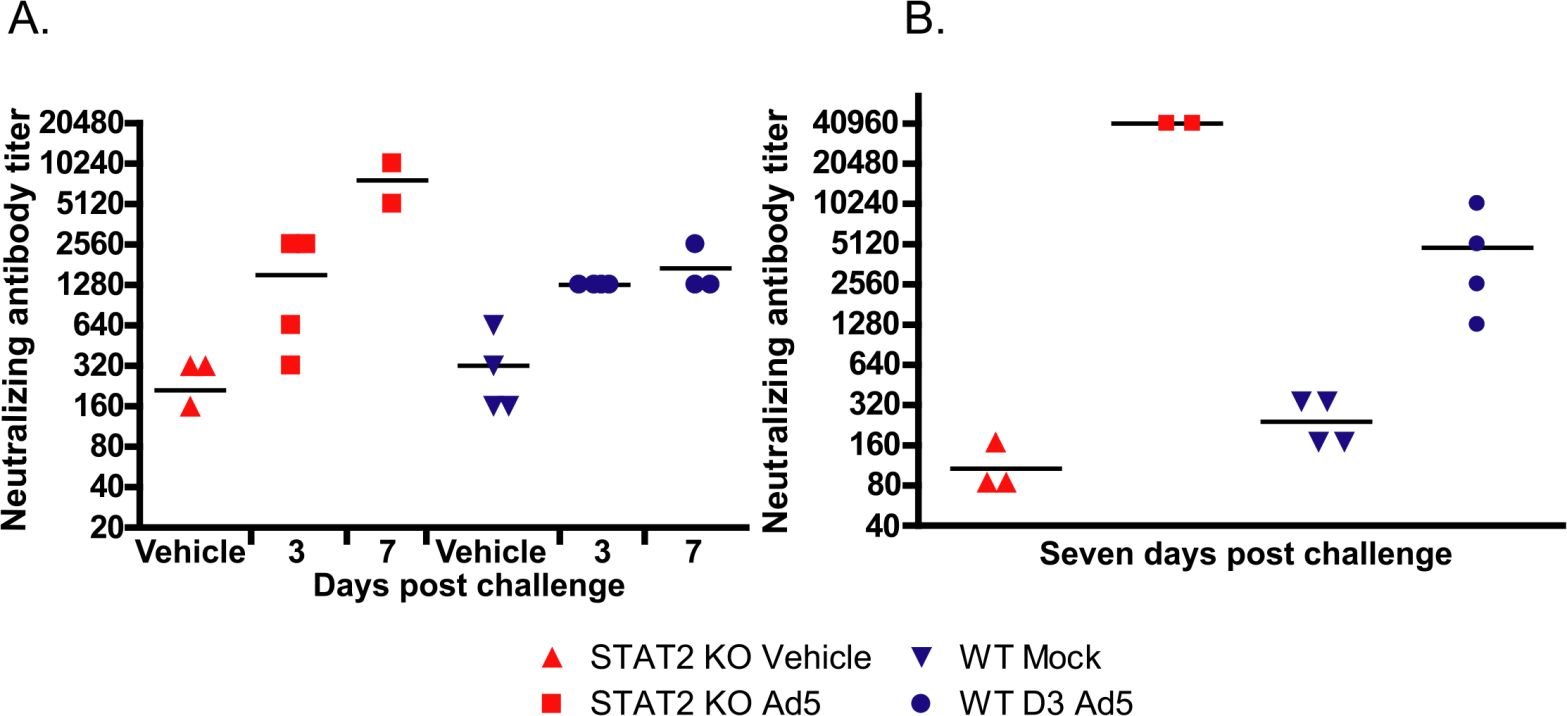
After Ad5 infection, the humoral immune response is accentuated in the STAT2 KO animals. Data from two parallel experiments (**A, B**) are presented. Reciprocal dilution of the serum that causes a 50% inhibition of Ad5 replication in cell culture is shown. Symbols denote data from individual animals. Data from moribund animals were excluded.

Neutralizing antibody (NAb) levels were determined in serum collected at sacrifice. For both STAT2 KO and wt hamsters, NAb levels were elevated in response to Ad infection at 3 days post challenge (Fig 7A). However, for the STAT2 KO hamsters the NAb titers kept increasing after this time, while the NAb levels for wt hamsters reached a plateau by this time (Fig 7A). This finding was corroborated by data from another experiment, in which approximately 10-fold more NAb was raised in STAT2 KO hamsters by 7 days post challenge than in wt ones (Fig 7B).

**Fig 8:**
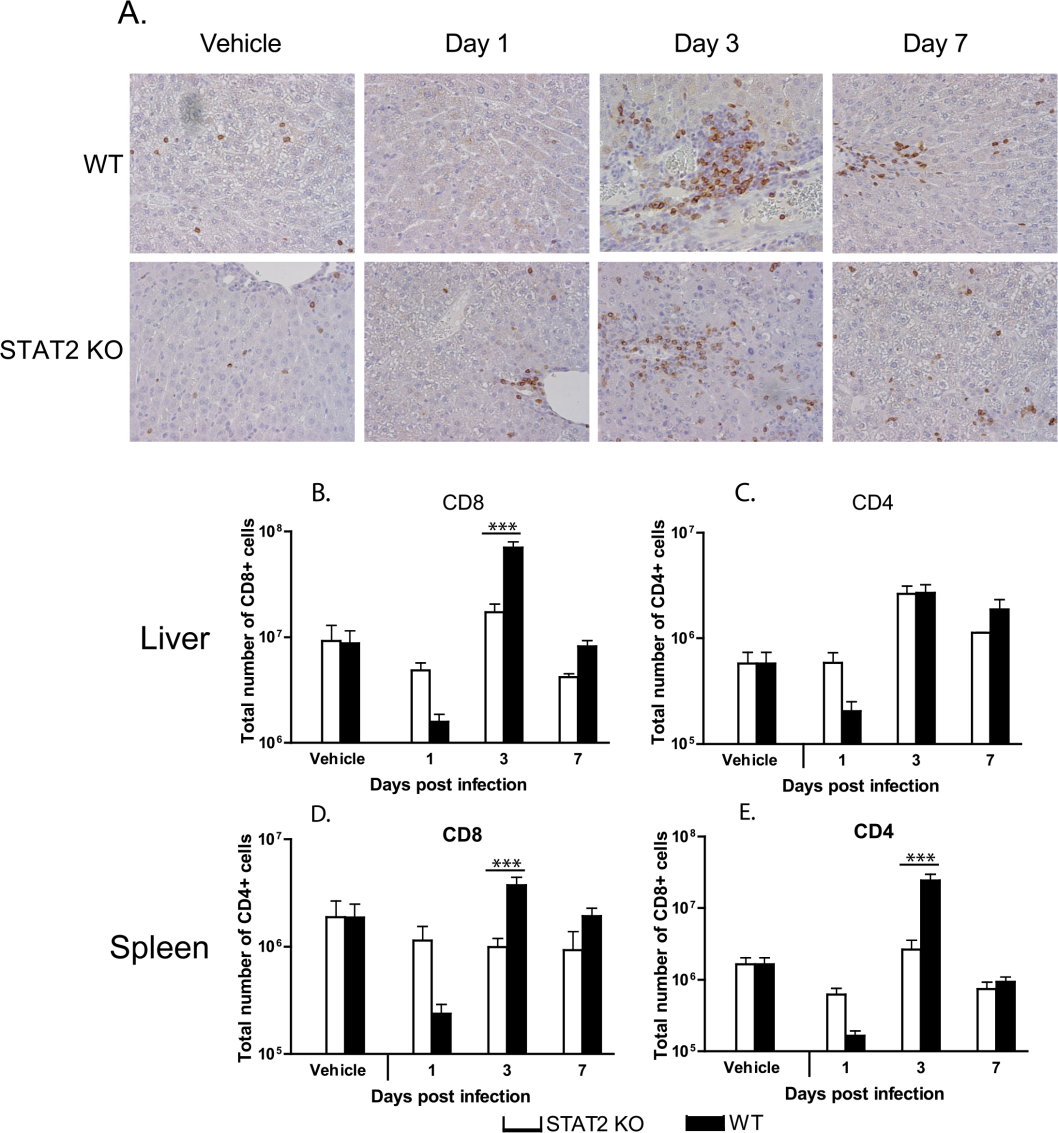
The cellular immune response to Ad5 infection is similar in wt and STAT2 KO hamsters. **A.** Immunohistochemical staining for hamster CD3. Flow cytometry for CD8+ (**B, D**) and CD4+ (**C, E**) T lymphocytes in the liver (**B, C**) and spleen (**D, E**) of Ad5-infected hamsters. The columns represent the mean; the error bars signify the standard error of the mean. n = 4, with the exception of D7 STAT2 KO, for which n = 2. ***: p<0.001.

When testing the liver for infiltrating T lymphocytes by immunohistochemical staining for CD3, we found that the baseline T-cell numbers in the liver were similar for both hamster strains; however, at 1 day post challenge, T-cell numbers in the liver dropped with wt hamsters, while perivascular infiltration had started with STAT2 KO hamsters by this time (Fig 8A). At 3 and 7 days post challenge, T-cell infiltration in STAT2 KO hamsters was similar to that in wt animals (Fig 8A).

We determined by flow cytometry the number and ratio of CD8+ (Fig 8B) and CD4+ (Fig 8C) T-cells infiltrating the liver, and found that in vehicle-treated hamsters there was no difference between the two hamster strains. At 1 day post challenge, there was a decrease in the number of both CD8+ and CD4+ T-cells in the liver of wt animals, while no such decrease was seen in the liver of STAT2 KO animals (Fig 8B and 8C). This finding corroborates the observation that the number of CD3+ cells declined in the liver of wt hamsters at 1 day post challenge (Fig 8A). A similar decrease in both CD8+ and CD4+ T-cell numbers was observed in the spleen of wt hamsters (Fig 8D and 8E). At 3 days post challenge, there was an increase in the number of both CD8+ and CD4+ T-cells in the liver of both strains of hamsters; however, there was a significantly larger number of CD8+ cells in the liver of wt animals compared to STAT2 KO hamsters (Fig 8B). Similar observations were made for the spleen (Fig 8D and 8E). At 7 days post challenge, the number of both populations of T-cells declined in the liver and spleen (Fig 8) of both hamster strains.

**Fig 9:**
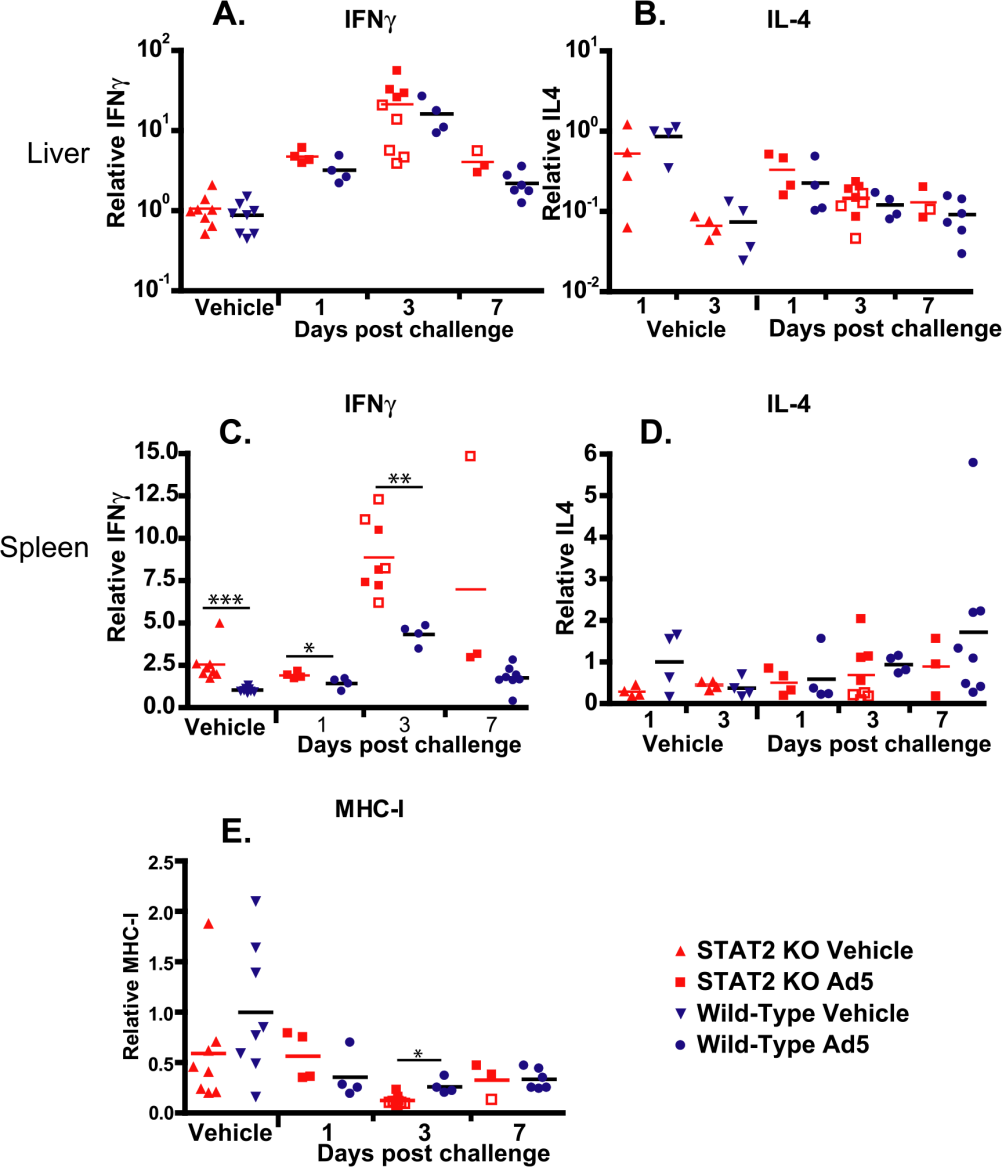
Cytokine expression in the liver is similar in wt and STAT2 KO hamsters; however, the expression of IFNγ is up-regulated in the spleen of STAT2 KO hamsters compared to wt ones. IFNγ (**A, C**) and IL4 (**B, D**) expression levels in the liver (**A, B**) and spleen (**C, D**), and MHC-I mRNA levels in the liver (**E**) are shown as fold changes in mRNA level. Symbols denote data from individual animals; empty symbols signify that the samples were collected from an animal that was sacrificed moribund. *: P<0.05; **: P<0.01; ***: P<0.001.

To test whether there was any difference between the number of Th1 and Th2 lymphocytes infiltrating the liver, we determined the changes of IFNγ and IL-4 transcripts. After challenge, IFNγ mRNA levels were elevated in the liver of both strains of hamsters; peaking at 3 days post challenge, and starting to decline at 7 days post challenge (Fig 9A). Samples collected from moribund STAT2 KO animals had significantly lower levels of IFNγ mRNA levels in their liver at 3 days p.i. (P = 0.0159; compare the open and closed square symbols) (Fig 9A). Conversely, a slight decrease over time after challenge was seen in IL-4 mRNA levels in the liver, with no significant differences between the two hamster strains (Fig 9B). In the spleen, baseline IFNγ mRNA levels were significantly higher in STAT2 KO hamsters than in wt ones, and reached significantly higher peak values at 3 days post challenge (Fig 9C). No significant changes were seen in IL-4 mRNA levels in the spleen (Fig 9D).

We have also determined the expression levels for the Class I major histocompatibility complex (MHC-I) mRNA in the liver. We found that at 3 days post challenge, there was a small but statistically significant decrease in the abundance of MHC-I mRNA in the liver of STAT2 KO hamsters compared to the wt ones (Fig 9E).

## Discussion

The immune response to human Ad infections has been studied extensively using mice, and valuable information was collected as to the response to the early phases of viral infection, i.e. virus attachment, entry, endosomal escape, etc. [8, 44, 45]. However, the experiments conducted in mice could not investigate the immune response induced by events in the late phase of infection, i.e. DNA replication, expression of late proteins, cell death and lysis, because human Ads replicate very poorly in mice. Our work is the first of its kind, using a permissive animal model to investigate the effect of Type I IFNs on the replication of, and the pathology induced by human Ads.

Ads, like other viruses, are believed to employ various mechanisms to thwart the effects of IFNs as indicated by studies in cell culture [1, 5]. It was demonstrated that the Ad VA RNA I effectively inhibits the action of PKR in cell culture [39], thus abrogating the antiviral effect of IFNα. As anticipated, we have shown that the replication of a mutant Ad deleted for the VA RNA I gene is inhibited in IFNα-treated primary hamster kidney cells, while there was only a modest suppression of the replication of a wt Ad by IFNα (Fig 5).

Ad has other mechanisms to suppress the function of Type I IFNs. E1A expression inhibits the expression of ISGs by suppressing the transcriptional co-activator p300 [46] and by suppressing the Jak-STAT signal transduction pathway [36, 47–50]. In Ad-infected cells, E1A was reported to overcome the IFN-mediated inhibition of replication of vesicular stomatitis virus [51], the IFN-induced resistance to NK cell lysis [52], and to suppress Jak-STAT signaling [53, 54].

The Ad E1B-55K protein functions to inhibit the transcription of some ISGs as shown by studies in cells infected with Ad E1B-55K mutant viruses [55, 56]. E1B-55K expressed alone did not have this inhibitory function.

In studies with Ad mutants that do not synthesize the E4orf3 protein, IFN blocked viral DNA replication and early gene expression [57, 58]. The E4orf3 protein apparently exerts its anti-IFN function by causing the components of promyelocytic leukemia protein (PML) nuclear bodies (PML-NB) to rearrange into nuclear tracks [57]. PML-NBs, which contain a large number of cellular proteins, some of which are induced by IFN, function in multiple cellular responses including antiviral defense [59].

It is interesting and somewhat puzzling that in the studies discussed above, in which an Ad mutant lacks only one of the anti-IFN gene products, namely VA RNA I, E1A, E1B-55K, or E4orf3 but expresses the other gene products, treatment of infected cells by IFN has such strong anti-Ad effects. Many of these studies were conducted in cancer cell lines, some of which may have defects in IFN-induced effects. However, we observed this same phenomenon in our studies with *dl*331 (lacks VA RNA I) in primary hamster kidney cells.

Considering that Ad5 has at least 4 functions that are reported to counter IFN antiviral effects, it is somewhat surprising that we found that control of Ad5 replication by Type I IFNs is of such high importance *in vivo* in the Syrian hamster model. In the liver of STAT2 KO hamsters, human Ad5 replicated to a 100- to 1000-fold higher titer compared to their wt counterparts (Fig 2), and the virus load was also much higher in the lung and kidney of the STAT2 KO animals (Fig 3). As might be expected, this high virus load resulted in higher morbidity and mortality (Fig 1A). As to the reasons for this elevated Ad replication, we have shown that the Type I IFN signal transduction pathway is not functional in STAT2 KO hamsters; these animals cannot up-regulate the expression of ISGs upon virus infection (Fig 4). In the infected cells, this failure to up-regulate the expression of ISGs will most likely lead to increased virus replication. Further, in the STAT2 KO animals, the immunomodulatory function of Type I IFNs may be impaired as well. At 3 days post challenge, we found less infiltrating NK cells in the livers of Ad5-infected STAT2 KO hamsters than in the livers of wt ones (Fig 6A), though the levels of IFNγ transcripts, one of NK-cells’ main antiviral product, were similar in the livers two strains of hamsters (Fig 9).

Another interesting finding pertains to the efficacy with which all surviving STAT2 KO animals cleared the virus. This, the abundance of neutralizing antibodies (Fig 7) and infiltrating CD3+ cells (Fig 8) in the liver indicate that the adaptive immune responses are intact. This is somewhat at variance with results presented by Zhu et al. that Type I IFN signaling on both B cells and CD4 T cells is critical for eliciting a neutralizing antibody response to replication-defective Ads in mice [60]. A possible explanation for this discrepancy may be that the adaptive immune response in a permissive model is probably stimulated by more factors, like the inflammation caused by the lysis of infected cells and the abundance of viral antigens than in a non-permissive one. The abundance of viral antigens (100- to 1000-fold higher virus load than wt hamsters in the livers and probably other organs) may be a factor in the very high neutralizing antibody levels seen with STAT2 KO hamsters. Although more CD8+ T cells infiltrated the liver of Ad5 infected wt hamsters than STAT2 KO animals (Fig 8), the IFNγ and IL-4 profile of the liver-infiltrating lymphocytes was identical for wt and STAT2 KO animals (Fig 9), thus it is not likely that a Th2-type skewing of the adaptive immune response is responsible for the elevated neutralizing antibody responses in STAT2 KO hamsters. The slight elevation of MHC-I mRNA levels in the liver of wt hamsters over their STAT2 KO counterparts (Fig 9E) might have resulted in better retention of infiltrating CD8+ T lymphocytes and thus might have contributed to the larger number of CD8+ cells seen in the liver of wt animals.

It was reported earlier that intravenous injection of large doses of Ads can lead to leukopenia both in humans, non human primates, and mice [61–63]. We have seen a similar phenomenon in wt hamsters: at 1 day post challenge, the number of CD3+ cells declined in the liver, as did the number of CD4+ and CD8+ cells in the liver and spleen (Fig 8). However, no similar decline in leukocyte numbers at 1 day p.i. was seen with STAT2 KO hamsters (Fig 8). This difference between wt and STAT2 KO animals suggests that the Type I IFN production in response to Ads causes leukopenia. This conclusion is supported by findings that Type I IFN treatment of mice and hepatitis C patients causes leukopenia [64–67]; thus, a similar mechanism may be at work during Ad infection.

By 3 days p.i., the number CD3+ lymphocytes rebounded with wt hamsters, and the number of CD8+ T cells infiltrating the liver, as well as the number of CD4+ and CD8+ T cells in the spleen were elevated compared to that of vehicle-treated hamsters (Fig 8). At 3 days p.i., a similar increase in the number of these lymphocyte subsets was observed with the STAT2 KO animals, albeit to a lesser degree (Fig 8). However, this difference does not result in a defect in clearing the virus infection (Fig 2).

This is the first time that data regarding the immune response with a STAT2 KO Syrian hamster, or indeed any kind of gene modified hamster, has been made available. We have verified that the Type I IFN pathway is disrupted with these animals, as evidenced by their inability to increase the expression of the ISGs PKR, OAS, and Mx2. We have characterized the immune response to Ad infection in these hamsters, and described the resulting pathology. We believe that this hamster strain is a valuable resource for researchers using Syrian hamsters as an animal model, and that our data will help investigators in their research. Further, our data highlight the importance of the Type I IFN response in controlling systemic Ad infections, and as such our results may have some clinical importance.

## Materials and Methods

### Cells and viruses

A549 human lung adenocarcinoma cells were purchased from the American Type Culture Collection (ATCC) (Manassas, VA), while HEK293 human embryonic kidney cells were purchased from Microbix (Mississauga, Ontario, Canada). Both cell lines were cultured in Dulbecco’s modified Eagle’s medium (Sigma-Aldrich, St Louis, MO, USA) with 10% fetal bovine serum (FBS) (complete DMEM) at 37°C. Ad5 *wt*500, a wt human Ad5 isolate, was derived in our laboratory by plaque purification from an Ad5 stock purchased from ATCC. The *dl*309 virus [68] was obtained from Elizabeth Moran; *dl*331 [69] was obtained from Tom Shenk. *dl*331 contains a deletion that blocks expression of VA RNA I, while *dl*309 is considered to be the phenotypically wild-type parental virus for *dl*331. The titer of the viruses was determined by plaque assay.

### Animals

The hamster strain (STAT2 KO) homozygous for a +1 frameshift mutation in the N-terminal domain of the STAT2 gene was reported previously [34]; wt hamsters were purchased from the same supplier from which the parental hamsters of the STAT2^-^ strain originated (Charles River Laboratories, Wilmington, MA). With a genomic PCR assay developed in house, both hamster strains were found to carry a latent hamster polyoma virus infection [70]. In some of the STAT2 KO animals this infection resulted in tumors (hemangiomas and lymphomas). All animals with lymphomas and ones with large hemangiomas were excluded from our study. All studies were approved by the Institutional Animal Care and Use Committee of Saint Louis University and were conducted according to federal and institutional regulations.

### Infection of hamsters with adenovirus

Two groups of hamsters were established; one with STAT2 KO animals (10 males and 14 females) and the other with wt ones (females only). There were 24 hamsters in both groups. For the STAT2 KO animals, male and female hamsters were equally distributed to all treatment groups. The animals were anesthetized with a ketamine/xylazine mixture, and PBS or Ad5 was injected i.v. (via the jugular vein). Half of the animals in both the STAT2 KO and wt groups received vehicle (PBS), while the remaining hamsters were injected with 2x10^11^ plaque forming units (PFU)/kg of Ad5. Four Ad5-infected and 4 vehicle-treated hamsters from both groups were sacrificed at 1, 3 and 7 days after challenge. The body weights and signs of morbidity of the animals were recorded daily. Hamsters that became moribund before Day 7 were sacrificed as needed. Besides animals judged moribund by observation, we sacrificed all hamsters that lost more than 20% of their original body weight.

By Day 4 post challenge, we lost all the Ad5-infected STAT2 KO animals scheduled to be sacrificed at 7 days post challenge. To compensate for this loss we altered the design of the study, and injected the remaining 4 wt and 4 STAT2 KO vehicle control animals (no procedures were performed on these hamsters at this point) with 1.6x10^11^ PFU/kg of Ad5 with the intent of sacrificing these hamsters at Day 7. However, 2 of the 4 STAT2 KO hamsters died at Day 4, thus, we had only two animals in this group for the Day 7 sacrifice time point. Data obtained from animals sacrificed moribund at Days 3 and 4 were grouped with the data collected from animals sacrificed according to schedule at 3 days post challenge, while data collected from a single hamster sacrificed moribund at 6 days post challenge were grouped with data from the group sacrificed at 7 days post challenge. All data collected from moribund animals is clearly marked when used.

### Necropsy, histopathology and clinical pathology

At necropsy, the animals were bled out and liver, lung, and kidney was collected. Virus was extracted from the liver and was quantified by the 50% tissue culture infectious dose (TCID_50_) assay in HEK293 cells as described previously [30]. A portion of the collected tissues was preserved in formalin and processed for histopathology (Seventh Wave Laboratories, St. Louis, MO). Immunohistochemical staining was performed by the Histopathology and Tissue Shared Resource of Georgetown University, using 1:1000 dilution of the Adenovirus Ab-4 (4D2) (Lab Vision, Fremont, CA) and 1:200 dilution of the CD3-ε (M-20) (Santa Cruz Biotechnology, Santa Cruz, CA) antibodies to stain for the Ad fiber and hamster CD3 proteins, respectively. Serum was assayed for alanine transaminase levels (Advanced Veterinary laboratory, St. Louis, MO). The serum concentration of hamster IFNα was determined using a hamster IFNα ELISA kit (MyBiosource, San Diego, CA), according to the manufacturer’s instructions.

### Determining the relative abundance of mRNA for various genes using reverse transcriptase-quantitative polymerase chain reaction (RT-qPCR)

Total RNA from liver and spleen was extracted from each hamster by homogenizing a fraction of collected tissues in RNALater buffer (Qiagen, Valencia, CA) and then extracting the RNA using the RNeasy mini kit (Qiagen). All RNA samples were treated with RNase-free DNase followed by RNA cleanup to eliminate DNA contamination. The RNA yield was determined on a NanoDrop-2000 spectrophotometer.

For RT-qPCR, two micrograms of each RNA and 50 pM of oligo(dT) primer were used for *in vitro* reverse transcription (RT) using High Capacity cDNA Reverse Transcription kit (ABI, Forster city, CA). SYBR-green based qPCR was used to specifically detect target gene mRNA. Primer sequences for PKR, Mx2, IFN-γ, and IL-4 were described previously [71]. The primers for OAS-3 (F, 5’-AGGTGCTTAAGGTGGTTAAGGG-3’; R, 5’-TGCTCAGAGAAGTGCTGGAAG-3’), ribosomal protein S6 kinase polypeptide1 (RPS6KB1) (F, 5’-TCAGACCGGTGGAAAACTCTAC-3’; R, 5’-TGATGCAAATGCCCCAAAGC-3’), and KiRL1.1 (F, 5’-CCTTTACTCTTGCTGCTATGC-3’; R, 5’-TTTGACTCTTGATCCCTGTTG-3’) were designed using Primer3 software (Whitehead Institute for Biomedical Research, Cambridge, MA) and synthesized by Integrated DNA Technologies (Coralville, IA). The PCR was set up in a 20 μl volume containing 1x SYBR select master mix (ABI), 250 nM forward and reverse primers, and 3 μl of the diluted RT template. Quantification was done in triplicate for each sample using an ABI model 7500 genetic analyzer with the following cycling parameters: 1 cycle at 50°C for 2 min, 1 cycle at 95°C for 10 min, followed by 40 cycles of 95°C for 15 sec and 60°C for 1 min. Dissociation curve analysis was performed following PCR to verify the specificity of the amplified product.

The data were analyzed using the ΔΔCt method. Housekeeping gene RPS6KB1 was used as an endogenous control for normalization. Briefly, the Ct of each target gene in a treated hamster was first normalized to the Ct of the endogenous control (ΔCt) and then compared to the same normalized gene in a mock-treated (calibrator) hamster to determine the ΔΔCt. The final value is displayed as the relative fold change between the Ad5-infected and mock-treated hamsters.

### Determining the yield of infectious virus after treatment of primary hamster kidney cells with IFNα in cell culture

To generate primary hamster kidney cells, kidneys were collected from wild-type and STAT-2 KO hamsters. Kidney tissue was minced and trypsinized. The material was passed through a mesh sieve to give a cell suspension. Kidney cells were centrifuged and plated onto cell culture dishes in DMEM containing 10% FBS. After 4 days, cells were trypsinized and plated into 12-well plates at 8x10^4^ cells per well. After an additional 5 days, cells were left untreated or treated with 250 or 1000 U/ml of human IFNα (PBL Assay Science; cat. #11105–1) for 24 h, and then infected with 25 PFU/cell of *dl*309 or *dl*331. Cell numbers at infection were 1.29x10^5^ cells/well (wild-type kidney cells) or 8.39x10^4^ (STAT-2 KO kidney cells). Infections were done in duplicate for each IFNα concentration; cells remained in IFNα during and after infection. Monolayers were washed at 24 h p.i. and 1 ml DMEM/10% FBS was added per well. Cells were freeze-thawed and harvested. Samples were sonicated for 4 min. Infectious virus yield was determined by TCID_50_ assay on HEK 293 cells.

### Determining the number of CD8+ and CD4+ cells in the liver and spleen using flow cytometry

Liver and spleen were collected into complete DMEM and stored on ice until processing. Single cell suspensions were made by forcing the organs through a 100 μm cell strainer (Fisher Scientific, Waltham, MA) using the blunt end of a syringe plunger. The cell suspensions were washed in PBS. A leukocyte-enriched preparation of the liver homogenate was produced as described before [72]. Briefly, an equal volume of the liver suspension was layered onto room temperature Ficoll-Paque Plus gradient (GE Healthcare, Little Chalfont, United Kingdom) and centrifuged for 30 min with no brake. The supernatant enriched in white blood cells was collected, diluted with PBS 1:1, pelleted, and washed in PBS. The cell pellets were re-suspended in a final volume of 300 μl of PBS. For the spleen, single cell suspensions were pelleted as described above, and re-suspended in 7 ml Pharm Lyse buffer (Beckton Dickinson, Franklin Lakes, NJ). The samples were incubated for 15 min at room temperature and then diluted 1:1 with PBS containing 2% fetal bovine serum (FACS buffer). The samples were then washed in DPBS, and the cell pellets were re-suspended in a final volume of 300 μl PBS. All cell suspensions were transferred to 96 well tissue culture plates, and kept on ice until staining.

For staining, the plates centrifuged at 1200 rpm for 5 min, and the supernatants were discarded. The cells were incubated with Live/Dead Fixable Aqua (Life Technologies, Grand Island, NY) cell stain for 30 min in the dark and on ice. The cells were washed twice with FACS buffer and then re-suspended in FACS buffer containing fluorochrome-conjugated antibodies. Cells were stained with a 1:2000 dilution of PE conjugated anti-mouse/rat MHC class II (I-Ek; clone 14-4-4S), a 1:200 dilution of FITC conjugated anti-rat CD8b (clone 341) and a 1:200 dilution of A700 conjugated anti-mouse CD4 (clone L3T4) (all from E-Bioscience, San Diego, CA). After 1 h incubation, the plates were washed twice with PBS, and the cell pellets re-suspended in 2% paraformaldehyde solution. After 5 min incubation, the cells were washed in PBS and re-suspended in FACS buffer. The samples were stored at 4°C for less than 24 h before being analyzed on a BD Biosciences LSR II. The data was acquired using FACSDiva software (BD) on the LSR II, and the data analysis was done at a separate computer using FlowJo.

### Determining the anti-Ad5 neutralizing antibody (NAb) titers in the serum

Serum samples were incubated for 30 min at 56°C to inactivate complement. Serum samples (in four replicate wells) were diluted twofold in a 96-well plate in complete DMEM. Ad5 was added to the dilutions of sera at 100 PFU per well, and after 1 h incubation at 37°C with the virus, A549 cells were added to each well at 5x10^5^ cells per plate. The plates were incubated at 37°C for 10 days, after which live cells were stained with neutral red (30 mg/ml in PBS). The cell-bound dye was extracted with 100 ml of acidified ethanol solution (50% ethanol and 1% acetic acid in H2O), and the absorbance was measured at 550 nm on a Synergy 4 microplate reader (BioTek, Winooski, VT, USA). The NAb titer was calculated as the reciprocal dilution causing 50% inhibition of viral cytopathic effect.

### Statistical analysis

Statistical analysis was performed using GraphPad Prism (version 4) software (GraphPad Software, La Jolla, CA). The overall effect was calculated using the Kruskal-Wallis test, and comparison between groups was performed using the Mann-Whitney U test. *P* values of 0.05 were considered significant.

### Ethics statement

All animal studies were approved by the Institutional Animal Care and Use Committee of Saint Louis University (protocol# 2015). The studies were conducted according to regulations in the Animal Welfare Act, the PHS Policy on Humane Care and Use of Laboratory Animals, and according to the recommendations of the Guide for the Care and Use of Laboratory Animals.
