# Correction: STAT2 Knockout Syrian Hamsters Support Enhanced Replication and Pathogenicity of Human Adenovirus, Revealing an Important Role of Type I Interferon Response in Viral Control

**DOI:** 10.1371/journal.ppat.1005392

**Published:** 2016-01-08

**Authors:** Karoly Toth, Sang R. Lee, Baoling Ying, Jacqueline F. Spencer, Ann E. Tollefson, John E. Sagartz, Il-Keun Kong, Zhongde Wang, William S. M. Wold

The following information is missing from the Funding section: This work was also partially supported by the Next-Generation BioGreen21 Program (Grant No. PJ01107704), Rural Development Administration, Republic of Korea (to Z.W.).
